# We have liftoff: A discovery study to use artificial intelligence to identify adaptative profiles for future space missions

**DOI:** 10.14814/phy2.70447

**Published:** 2025-08-29

**Authors:** Barbara Le Roy, Damien Claverie, Lucas Sauvadet, Charles Martin‐Krumm, Marion Trousselard

**Affiliations:** ^1^ Human Adaptation Institute Marseille France; ^2^ Stress Neurophysiology Unit French Armed Forces Biomedical Research Institute Brétigny‐sur‐Orge France; ^3^ Pony Industries Clermont‐Ferrand France; ^4^ University of Lorraine, Inserm, INSPIIRE UMR 1319 Nancy France; ^5^ École de Psychologues Praticiens, Catholic Institute of Paris, EA Religion, culture et société Paris France

**Keywords:** adaptation, classification, health, machine learning, space analogs

## Abstract

Long‐duration space missions will challenge astronauts' adaptive capacities. Interoception and heart rate variability (HRV), reflecting parasympathetic activity, are increasingly recognized as predictors of adaptation and health. This study investigated whether artificial intelligence may predict adaptation profiles from interoceptive and HRV responses accross different space analogs. Data were collected from 84 participants in four environments: parabolic flight, nuclear submarine, sea survival simulation, and chemical/biological hazard exercises. Interoceptive sensitivity and HRV were measured to identify adaptation profiles using clustering. Baseline data were then used to train a support vector machine (SVM) to predict these profiles. Three adaptation profiles emerged, differing in interoceptive awareness, body–mind integration, and neuroception. The SVM model predicted these profiles with 79% accuracy. These findings demonstrate the feasibility of using machine learning to anticipate adaptation outcomes based on physiological and interoceptive markers. They emphasize the embodied nature of adaptation and the relevance of interoceptive pathways in HRV dynamics. This work provides new directions for optimizing astronaut training by tailoring preparation to individual physiological profiles. Tomorrow is here, we are ready for take‐off.

## 1 INTRODUCTION

Upcoming long duration space exploration (LDSE) programs mean that it is essential to minimize the risk to astronaut crews. The literature on space, and space analog environments highlights psychiatric, psychological, cognitive, physiological, neurophysiological, sensory, and post‐mission impacts that are not only non‐linear over time, but also both pathogenic and salutogenic. The findings reported in the literature underline the need for countermeasures to improve adaptation (Le Roy, Martin‐Krumm, et al., 2023), as living in space is going to be a challenging endeavor (Faerman et al., 2023; Musso et al., 2018). These challenges include sensorimotor deconditioning, immune system alterations, circadian rhythm disruption, cardiovascular deconditioning, and heightened psychological stress, which can compromise mission success if not properly addressed. *Adaptation*, understood as the ability of a person to adjust in response to changing conditions or situations (VandenBos, 2007), is one of the five characteristics identified by NASA as necessary for future long‐distance space travelers (Landon et al., 2016), and some individuals will adopt their own combination of adaptive strategies (Le Roy, Martin‐Krumm, et al., 2023). These reactions help the person to reach a state of balance in response to new environmental constraints. Human adaptability to space environments and space analogs is, therefore, a key topic.

Studies have shown that the stress response, especially long‐term exposure to stressors, causes an alteration in central nervous system (CNS) signals, notably those regulated by the autonomous nervous system (ANS). This concept is in line with the notion of neuroception, which refers to the neural process by which the ANS assesses the degree of safety or threat contained in the environment (Porges, 1995; Porges, 2007). A set of physiological and behavioral regulatory loops is activated in response to environmental changes. *Homeostasis* refers to attempts by the organism to maintain its internal environment in a constant state of balance, while *allostasis* considers its variability (Logan & Barksdale, 2008; McEwen, 2004). Allostasis describes how the organism remains stable in the face of change, via regulatory mechanisms that ensure the independence of the internal environment from the external environment (Corcoran & Hohwy, 2018).

In recent work, the genomics, environment, vagus nerve, social interaction, allostatic regulation, and longevity (GENIAL) model developed by Mead and collaborators (Mead et al., 2020) appears to capture the evolution towards pathology, and how to promote health and longevity. One of the regulators is the vagus nerve, indexed by heart rate variability (HRV). HRV has long‐been considered as a marker of adaptation, and an efficient indicator of health (Laborde et al., 2017). Higher parasympathetic HRV is associated with better executive function, stress management, and social engagement (Beauchaine & Thayer, 2015). It has been found to be indicative of increased adaptive abilities (Laborde et al., 2017; Le Roy, Martin‐Krumm, et al., 2023; Porges, 2007; Schulz & Vögele, 2015), and improved health outcomes (Ernst, 2017; Kim et al., 2018; Thayer et al., 2012).

In this context, the vagus nerve may be viewed as a constant brake that is released when the sympathetic nervous system is activated, leading to different adaptive behaviors (Porges, 1995). Kashdan and Rottenberg (Kashdan & Rottenberg, 2010) argue that it serves as an indicator of psychological flexibility, which is essential for health. Moreover, other evidence suggests that interoceptive pathways contribute to the stress response due to ongoing, bidirectional communication along the brain–body axis, and through the activation of brain structures such as the nucleus tractus solitarius, the thalamus, the anterior cingulate cortex, and the insula (Schulz & Vögele, 2015).

*Interoception* refers to the process by which the CNS senses, interprets, integrates, and regulates signals originating from within the body, “providing a moment‐by‐moment mapping of the body's internal landscape across conscious and unconscious levels” (Khalsa et al., 2018), p. 1. Mehling and collaborators (Mehling et al., 2012; Mehling et al., 2018) were the first to develop a scale to examine subjective interoception through body awareness (the Multidimensional Assessment of Interoceptive Awareness questionnaire, MAIA). Individuals with a high level of interoception, as assessed by the MAIA, have been found to be able to manage acute stressful situations more effectively (Herbert et al., 2021; Schultchen et al., 2019), and have more positive health outcomes (Khalsa et al., 2018). Interoception therefore appears to ensure homeostatic and allostatic adaptation via improved self‐regulation of information about the person's internal physiological state (Berntson & Khalsa, 2021; Craig, 2002; Schultchen et al., 2019). In a review, Pina and collaborators (Pinna & Edwards, 2020) highlighted that both HRV (especially the parasympathetic branch of the ANS), and interoception improve emotional regulation strategies. Regulatory responses therefore appear to act at both the conscious level, through emotions and feelings, and at the autonomic level (Damasio & Carvalho, 2013).

Although LDSE astronaut crews will be well‐trained and well‐prepared, they remain human. While they may have the resources to cope with most hazards, what they might encounter cannot be predicted. No place on Earth is sufficiently extreme to replicate all of the stressors that could be encountered during an LDSE mission. The best comparisons are isolated and confined environments, which may also be extreme and unusual; these environmental constraints are replicated in space analogs (Le Roy, Martin‐Krumm, et al., 2023). Space analogs are known to provide unique opportunities to study human adaptation in ecological environments, where life‐threatening risk is pervasive.

Typical examples include polar stations, space simulation facilities, and sub‐surface nuclear‐powered submarines (SSBN). They share similar environmental stressors and mission requirements, such as rigorous selection procedures, carefully selected crew, high‐technology facilities, the mission profile, and crew skills. While data from participants in professional or experimental settings cannot be compared to data from astronauts during a space mission, they are nevertheless an opportunity to expose crews to some stressors for a specific period of time, and thus to approximate the ecological space environment. However, most studies in space analogs rely on a small sample, making it difficult to characterize and generalize an adaptation profile. Finally, the literature suggests that psycho‐biophysiological and social variables are key factors.

The emergence of artificial intelligence (AI) may offer a way to better‐understand and predict which individuals are most at risk during a mission in these unusual environments. AI is already at the forefront of medicine, as real‐time monitoring is used to feed algorithms. Machine learning techniques provide a new way to understand human behavior, and make it possible to mine structured knowledge from an extensive dataset. These techniques appear to be well‐suited to addressing many of the challenges that will be encountered in the upcoming era of precision space medicine, notably the ability to predict the profile of crew, gain an insight into their adaptation strategies, and prepare training programs and countermeasures. Specifically, supervised machine learning algorithms such as support vector machines (SVM) may be able to analyze complex psychophysiological datasets, and identify hidden patterns in baseline measures. Therefore, in this study, we applied an SVM to baseline HRV and interoception data to predict adaptation profiles in space analog environments. To the best of our knowledge, this is a novel approach to both individual risk profiling, and resilience assessment.

The general objective of this pilot study is to investigate if machine learning analyses can help in understanding whether interoceptive and HRV factors can predict adaptation to a space mission. It builds upon several studies that have explored change in interoceptive and parasympathetic autonomic variables in healthy participants during experimental space analog missions. Our first aim is to define adaptation profiles based on mind–body changes before and after a space analog mission. In a second step, we seek to predict adaptation profiles based on pre‐mission mind–body functioning.

## 2 METHODS

### 2.1 Participants and data collection

We draw upon data from experiments conducted in four space analogs, and approved by a Committee for the Protection of Individuals. The first (ENACT) concerns parabolic flight in microgravity, an extreme and unusual environment (EUE) (ID‐RCB: 2022‐A01317‐36, Committee for the Protection of Individuals Ile‐de‐France XI) (Le Roy et al., 2025). The second concerns a two‐month mission in an SSBN, an isolated and confined environment (ICE) (ID‐RCB: 2017‐A01329‐44, Committee for the Protection of Individuals Sud‐Est VI) (Le Roy, Aufauvre‐Poupon, et al., 2023). The third (RAD'LÔ) concerns a simulation of survival at sea (ID‐RCB: 2017‐A01329‐44, Committee for the Protection of Individuals Sud‐Est VI) (Le Roy, Martin‐Krumm, et al., 2024), and the fourth (ANTIDOTE) concerns a medical intervention following a simulated chemical, biological, radiological, or nuclear (CBRN) attack. In the latter case, participants wore protective equipment that could be compared to an astronaut's extravehicular suit (ID‐RCB: 2021‐A03057‐34, Committee for the Protection of Individuals Ile‐de‐France XI) (Le Roy, Giaume, et al., 2024; Giaume et al., 2024). RAD'LÔ and ANTIDOTE are both ICE and EUE. The experiments were run between 2017 and 2022, and data were accessed between June 1, 2023 and July 31, 2023. Written consent was obtained for each study.

The dataset consisted of 84 participants and 16 variables, as follows. ENACT: 17 healthy scientists, including researchers (*n* = 6), physicians (*n* = 5), academic coordinators (*n* = 3), engineers (*n* = 2), and other (*n* = 1); SSBN: 19 male submariners belonging to the crew of the French vessel *Le Triomphant*; RAD'LÔ: marines working for the French Maritime Academy (*n* = 15), firefighters from the Marseille Naval Fire Battalion (*n* = 2), and volunteers from the Camondo Design School (*n* = 4); and ANTIDOTE: 27 healthy firefighters working for the Paris Fire Brigade.

Socio‐demographic characteristics of participants for each analog are described in Table 1.

**TABLE 1:** Socio‐demographic characteristics of participants.

Measurement	ENACT	SSBN	RAD'LÔ	ANTIDOTE
*N*	17	19	21	27
*M*_age_	41.35 ± 8.95	29.73 ± 6.94	22 ± 6.3	31.90 ± 7.0
*M*_height_	177.35 ± 6.83	NA	178 ± 9.41	175.00 ± 0.00
*M*_weight_	69.71 ± 9.06	75.78 ± 8.77	71.1 ± 13	75.51 ± 13.85
Gender (women/men)	23.52/76.47%	0/100%	33.33/66.66%	22.22/77.77%
In couple/with children	100/52.94%	78.94/NA	38.09/9.52%	88.88/37.03%
Contraception	25.00%	NA	28.57%	14.81%
Clinical medical history (i.e., hip replacement, deafness, pneumothorax)	11.76%	NA	4.76%	4.76%
Right‐handed/left‐handed	NA	100/0%	80.95/19.04%	NA
Personal major stressful events	2.66 ± 1.37	8.00 ± 0.50	NA	1.5 ± 0.83
Professional major stressful events	6.00 ± 5.04	5.00 ± 0.45	NA	2.33 ± 1.21
Previous experience in extreme environments	35.29%	100%	NA	NA

*Note*: Mean and standard deviation are reported when necessary. Other figures show the ratio of the number of participants. NA indicates a missing value.

Abbreviations: ANTIDOTE, simulation of a medical intervention following a CBRN attack while wearing protective equipment; ENACT, parabolic flight in microgravity; RAD'LÔ, simulation of survival at sea; SSBN, nuclear powered submarine.

**FIGURE 1:**
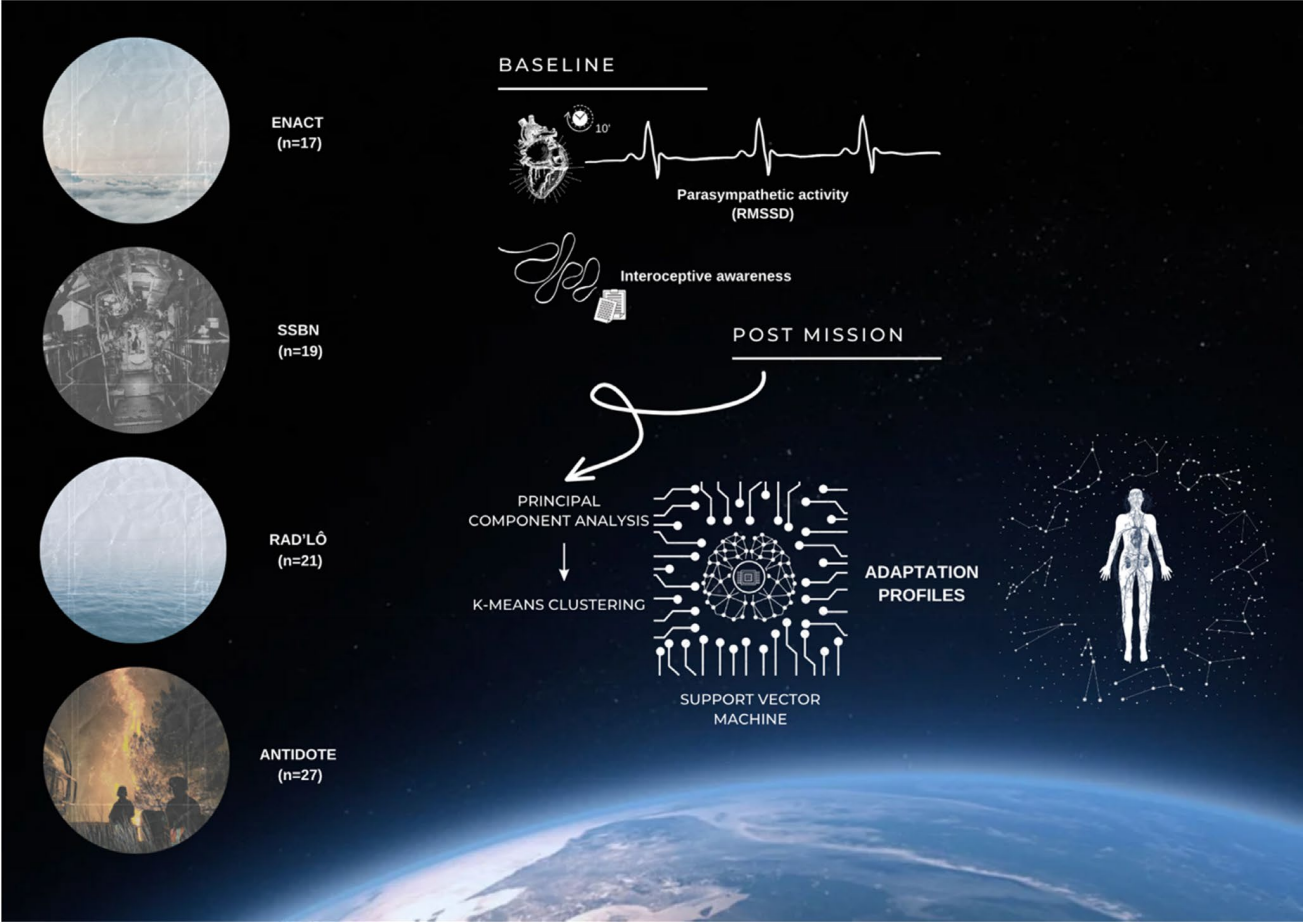
Experimental methodology.

Both interoceptive awareness and parasympathetic activity were recorded pre‐ and post‐mission. Data were analyzed to predict adaptation profiles, based on the person's pre‐mission psychophysiological state (Figure 1).

Table 2 presents the characteristics of the selected space analog stressors.

**TABLE 2:** Characteristics of space analog stressors.

Stressor	ENACT	SSBN	RAD'LÔ	ANTIDOTE
Extreme	‐	High	Very high	‐
Unusual	‐	High	Very high	High
Isolation	‐	~80 days	5 days	~2 h
Confinement	~4 h	~80 days	‐	~2 h
Length	~4 h	~80 days	5 days	~4 h
Ecology	Real	Real working environment	Real training	Simulation training
Stress	Acute	Chronic	Acute	Chronic
Sensory stimuli	High	Very low	Very high	Low
Threat	Low high	High	High	Low
Communication	Good	Complex	Complex	Complex

Abbreviations: ANTIDOTE, simulation of a medical intervention following a CBRN attack while wearing protective equipment; ENACT, parabolic flight in microgravity; RAD'LÔ, simulation of survival at sea; SSBN, nuclear powered submarine.

### 2.2 Variables and measurements

#### 2.2.1 Psychological measurements

An 18‐item sociodemographic questionnaire was used to collect general information on the participant's family situation, medical history, current health status, hobbies, and familiarity with extreme environments.

The 32‐item MAIA was used to evaluate interoceptive awareness. The scale is divided into eight sub‐factors that measure: awareness of uncomfortable, comfortable, and neutral body sensations (*noticing*); the response to sensations of pain and discomfort (*not‐distracting*, *not‐worrying*); the ability to regulate attention to body sensations and/or emotional states (*attention regulation*, *emotional awareness*, *self‐regulation*); and awareness of mind–body integration (*body listening*, *trusting*) (Mehling et al., 2012; Mehling et al., 2018).

#### 2.2.2 Physiological measurements

**FIGURE 2:**
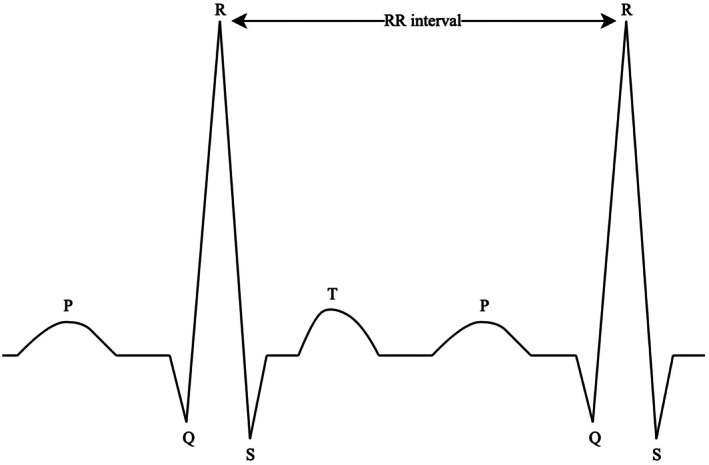
RR intervals extracted from the ECG.

An electrocardiogram (ECG) was recorded for a 10‐min period, to extract variation in the interval between two heartbeats (the RR interval), with participants in a sitting position (Figure 2). The HRV analysis followed guidelines reported in (Laborde et al., 2017; Task Force of the European Society of Cardiology and the North American Society of Pacing and Electrophysiology, 1996), which consider potential circadian variation, and used the *PyHRV* python library (Gomes et al., 2019). Raw ECG data were filtered using a finite impulse response band‐pass filter. R peaks were automatically detected using the *BioSPPy* python library (Carreiras et al., 2015). A Hamilton segmentation was performed on the filtered signal, followed by R‐peak correction with tolerance set to 0.05. R‐waves were manually examined to ensure correct detection. If an ECG sequence was overly noisy when visualizing the superposition of all QRS complexes, the time interval was manually removed to improve data quality. RR intervals were automatically detected with the *hrvanalysis* module using linear interpolation, and manually corrected for artifacts and ectopic beats. A detrended RR series was computed by subtracting the mean of RR intervals.

Time domain HRV metrics included the root mean square of successive differences (RMSSD) between adjacent RR intervals.

### 2.3 Data analyses

#### 2.3.1 Statistical analyses

Data analyses were performed with JASP (Amsterdam, version 0.16.3), an open‐source software package that is used for both classical and Bayesian analyses. The Shapiro–Wilk test was used to determine whether data were normally distributed. When the analysis was significant, effect sizes are reported. A one‐way ANOVA or the Kruskal–Wallis test (depending on the normality of the distribution) evaluated the interaction between adaptation profiles and features at baseline (pre‐mission).

The independent variable was composed of three profiles (classes 0, 1 and 2) highlighted by a *k*‐means clustering analysis. Dependent variables were the eight interoceptive measures (*noticing*, *not‐distracting*, *not‐worrying*, *attention regulation*, *emotional awareness*, *self‐regulation*, *body listening*, *trusting*), and the HRV metric (RMSSD), linked to parasympathetic activity. Holm post hoc analyses were performed when the *p*‐value was significant. Bayesian analyses were performed by applying equivalent analyses for the one‐way ANOVA. The Bayesian factor was calculated if no significant effect was detected. A low value provides support for the null hypothesis, and a high value indicates evidence in favor of the alternative hypothesis.

For all analyses, statistical significance was set at *p* < 0.05. A *p*‐value between 0.05 and 0.07 was considered as evidence of a trend.

#### 2.3.2 Principal component analysis

A principal component analysis (PCA) is a dimensionality reduction technique that is applied to a dataset in order to obtain new, latent variables. The purpose is to transform a dataset with potentially correlated variables into a new coordinate system, where the variables are uncorrelated and ranked by their importance in explaining the variance of the original data. This is achieved by projecting the data onto a set of new orthogonal axes in a high‐dimensional space that captures the maximum variance.

The starting point is a dataset with N data points, represented in a matrix X of size N x M, where each row $x_{i}$ represents a data point with M features. The PCA is structured as follows: the standardization of data $X_{\mathrm{std}}=\frac{X-\mu }{\sigma }$, where μ is the mean vector and σ is the standard deviation vector; the calculation of the covariance matrix, C = $\frac{1}{N}X_{\mathrm{std}}^{T}X_{\mathrm{std}}$; the computation of eigenvalues $\lambda_{i}$ and eigenvectors $V_{i}$; the selection of the top k eigenvectors corresponding to the k largest eigenvalues; and the projection of the data onto the lower‐dimensional space $X_{\mathrm{new}}=X_{\mathrm{std}}V_{\text{selected}}$, where $X_{\mathrm{new}}$ is the transformed data matrix in the reduced‐dimensional space and $V_{\text{selected}}$ contains the eigenvectors corresponding to the selected principal components. Finally, $\frac{\sum_{i=1}^{k}\lambda_{i}}{\sum_{i=1}^{M}\lambda_{i}}$ is the resulting explained variance, where k is the number of principal components selected, and M is the original number of features.

#### 2.3.3 The *k*‐means clustering algorithm

The goal of a clustering algorithm is to identify structure in a data distribution. The *k*‐means technique is a partitioning, non‐supervised machine learning algorithm where the goal is to learn from unlabeled data and extract similar patterns. In this case, $\vec{x_{n}}$ data are used, for which no desired $y_{n}$ values are available. Each cluster *C*_*k*_ is defined by its centroid *c*_*k*_, a vector of the input space, named the prototype. Any *x* is assigned to the *C*_*k*_(x) cluster, whose prototype is closest to x such that $k\left(x\right)=\text{ArgMink}\left(\text{dist}\left(x,c_{k}\right)\right)$. An Elbow curve, which is a heuristic method, was used to estimate the optimal number of clusters in the dataset when applying the clustering algorithm. More specifically, the optimal number is the number of clusters after which adding more clusters does not significantly reduce the within‐cluster sum of squares. A *k*‐means analysis was used to define the number of clusters characterizing our population, based on the factors obtained by the PCA.

#### 2.3.4 The support vector machine algorithm

A support vector machine (SVM) supervised machine learning algorithm is used to separate two classes from each other. The aim is to find a hyperplane that best divides data points belonging to different classes in a high‐dimensional space. The algorithm analyzes labeled data points, makes predictions, and classifies data into categories. In this case, $\vec{x_{n}}$ data are used together with the desired $y_{n}$ labels. The algorithm aims to maximize the margin (i.e., the minimum distance between any data point and the separating hyperplane) between data points of different classes. New data points can then be classified based on which side of the hyperplane they fall. The SVM approach is founded on two main principles: (1) optimizing linear separation by maximizing the margin between the hyperplane and each of the two classes; and (2) using a kernel function to transform the original input space into a higher‐dimensional space that is used to calculate the linear separation.

**FIGURE 3:**
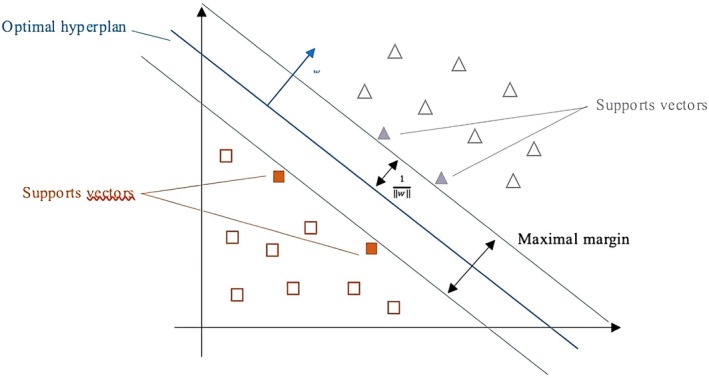
Illustration of the SVM algorithm.

Given a training dataset of N datapoints $\left(x_{1},y_{1}\right),\left(x_{2},y_{2}\right),\ldots ,\left(x_{N},y_{N}\right)$, where $x_{i}$ represents the feature vector of the $i^{\mathrm{th}}$ data point and $y_{i}$ is the corresponding class label, the SVM aims to find a hyperplane described by the equation: $h\left(\vec{x}\right)=\vec{w^{*}}.\;\vec{x}+b^{*}$, where ($\vec{w^{*}},b^{*})$ is the optimal solution to a constrained quadratic optimization problem (Figure 3).

The SVM algorithm is particularly useful when dealing with data that has multiple features or dimensions for the following reasons: it is less sensitive to outliers because it focuses on the support vectors that have the most impact on determining the hyperplane; it can handle non‐linear relationships between data points through the use of other kernel functions, allowing it to find complex decision boundaries; and it uses a regularization parameter to control the trade‐off between maximizing the margin and correctly classifying data points. Moreover, it can be applied to small samples (Zhang et al., 2019).

### 2.4 Algorithm implementation

#### 2.4.1 Missing data

The dataset has missing data for multiple reasons (e.g., mission constraints). In order to reduce noise due to missing data, a randomization function was applied. Here, the purpose was to introduce random noise to replace a missing value. If the value is not missing, the function returns the original value.

#### 2.4.2 Data normalization

Data were normalized using the *MinMaxScaler* function, which scales data to a specific range, named $x_{\text{scaled}}$, in this case between −1 and 1.

$$x_{\text{scaled}}=\frac{x-\min\left(x\right)}{\max\left(x\right)-\min\left(x\right)}$$

Here, the aim was to improve the numerical stability of the machine learning model.

#### 2.4.3 Feature selection

A PCA was applied to the normalized data, based on the delta of psychophysiological features (RMSSD and interoceptive awareness variables), denoted M, and defined as $\text{delta}=M_{\text{post mission}}-M_{\text{baseline}}$. Three principal components were identified that explained 80% of the total variance, and these components were used to estimate clusters of mind–body adaptation by applying a unsupervised *k*‐means algorithm and cluster labels.

Then, the SVM classifier was trained on the baseline dataset to identify which psychophysiological features contributed most to separating the clusters. The feature coefficients from the trained SVM model were extracted and stored in a list. These coefficients represent the importance of each feature in making classification decisions. Positive coefficients indicate that an increase in the value of the feature increases the likelihood of belonging to a certain class, while negative coefficients indicate the opposite. This process provides a better understanding of which features are most influential in distinguishing between clusters. The regularization parameter *C* was set to 1.0.

#### 2.4.4 Model training

The SVM classification was performed 1000 times, with different random training/ test splits. For each iteration, data were split into a training and a test set. Each iteration involved training the SVM model with a linear kernel on a random subset of the data and then evaluating its performance using a test subset. The model was trained on 50% of the total dataset (42 individuals) and tested on the remaining 50% (42 individuals). These proportions were chosen to ensure a sufficient number of examples in both subsets.

As the *k*‐means technique involves a multi‐class problem, each pair of classes was compared with the others to transform the problem into a series of binary classification tasks and predict $M_{\text{delta}}$ (change during the mission, and its role in stress adaptation). During training, the model tracks the best accuracy achieved during the loop, along with the corresponding random state that led to that accuracy. If better accuracy is found in any iteration, the best accuracy is updated to reflect the new optimal result, along with the random state that produced it. A cross‐validation was used to assess the generalization performance of the model, and a value of 10 was selected, due to the small dataset. The best model over the 1000 iterations was chosen.

#### 2.4.5 Validation and performance measurement

Several measures were used to evaluate the performance of the classifier. A confusion matrix assessed the performance of the SVM model on the test data and provided a detailed representation of true positives (TP), false positives (FP), true negatives (TN), and false negatives (FN). The classification generates a comprehensive report that includes sensitivity (the ability of the classifier to correctly identify non‐adaptive profiles), specificity (the ability of the classifier to correctly identify adaptive profiles), the F1‐score, and accuracy (the ratio of the total number of correct assessments to the total number of assessments) for each class.

Sensitivity is calculated as:

$$\text{Sensitivity}=\frac{\mathrm{TP}}{\mathrm{TP}+\mathrm{FN}}$$

Specificity is calculated as:

$$\text{Specificity}=\frac{\mathrm{TN}}{\mathrm{TN}+\mathrm{FP}}$$

F1 is calculated as:

$$F1=\frac{2\times \mathrm{TP}}{2\times \mathrm{TP}+\mathrm{FP}+\mathrm{FN}}$$

Accuracy is calculated as:

$$\text{Accuracy}=\frac{\mathrm{TN}+\mathrm{TP}}{\mathrm{TN}+\mathrm{TP}+\mathrm{FN}+\mathrm{FP}}$$

While the confusion matrix and the classification report provide insight into the model's ability to correctly identify different classes and its overall effectiveness, the learning curve highlights trends in model performance as a function of the size of the training dataset. It helps identify potential issues such as overfitting or underfitting, and provides a visual and quantitative assessment of the model's performance and accuracy.

**FIGURE 4:**
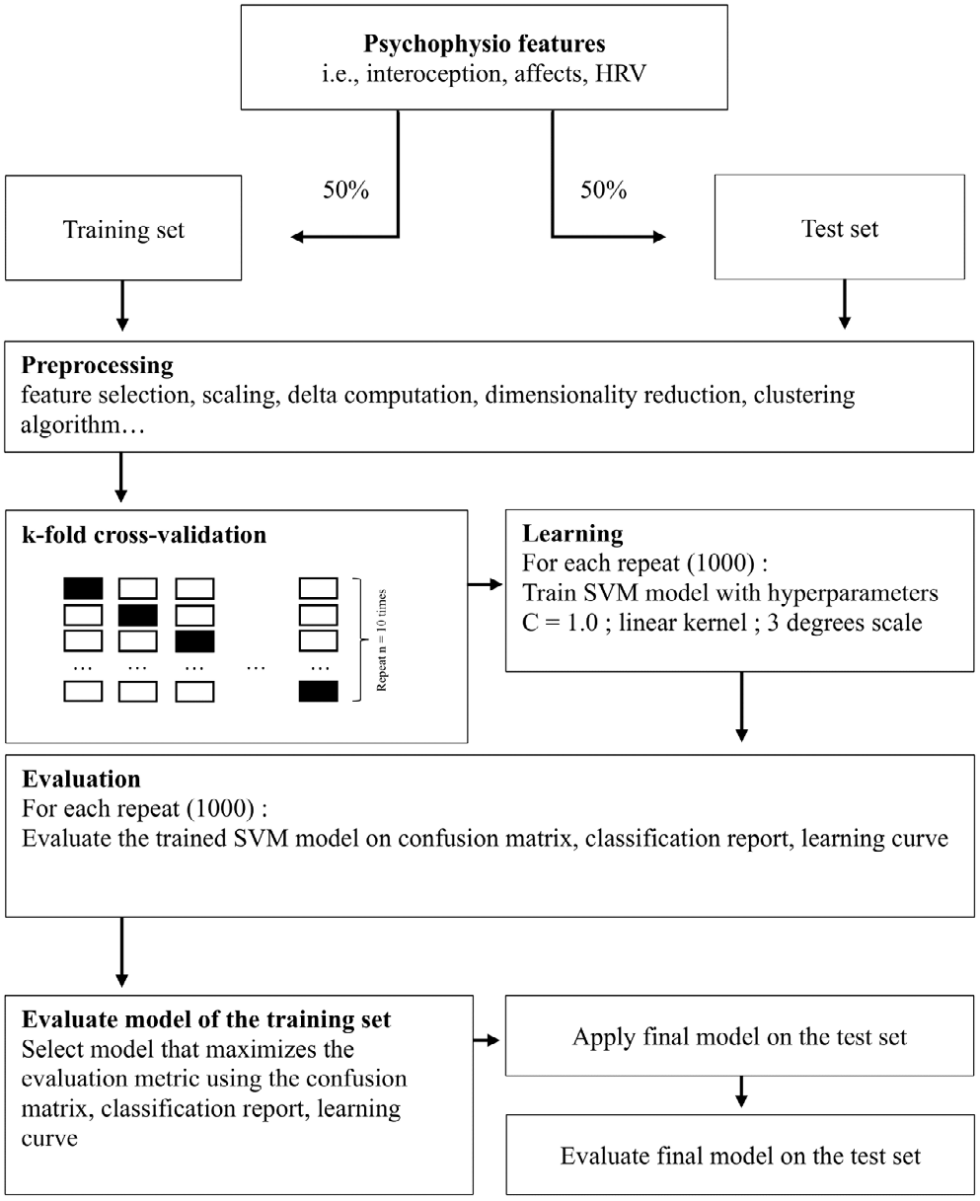
Schematic overview of the SVM model.

The architecture of the overall methodology is summarized in Figure 4.

## 3 RESULTS

### 3.1 Characterization of adaptive profiles at baseline

#### 3.1.1 Interoceptive awareness

No significant differences were found for any of the interoception subfactors. Results were as follows: *noticing* [*F* (2.81) = 0.019, *p* = 0.982, BF_10_ = 0.109]; *not‐distracting* [*F* (2.81) = 0.108, *p* = 0.898, BF_10_ = 0.117]; *not‐worrying* [*F* (2.81) = 0.176, *p* = 0.839, BF_10_ = 0.123]; *attention regulation* [*F* (2.81) = 0.117, *p* = 0.890, BF_10_ = 0.117]; *emotional awareness* [*F* (2.81) = 0.694, *p* = 0.503, BF_10_ = 0.186]; *self‐regulation* [*F* (2.81) = 1.867, *p* = 0.161, BF_10_ = 0.476]; *body listening* [*F* (2.81) = 0.836, *p* = 0.437, BF_10_ = 0.206]; and *trusting* [*F* (2.81) = 0.119, *p* = 0.888, BF_10_ = 0.118].

### 3.2 HRV

No significant difference was found for RMSSD [F (2.81) = 0.647, *p* = 0.526, BF_10_ = 0.180].

**FIGURE 5:**
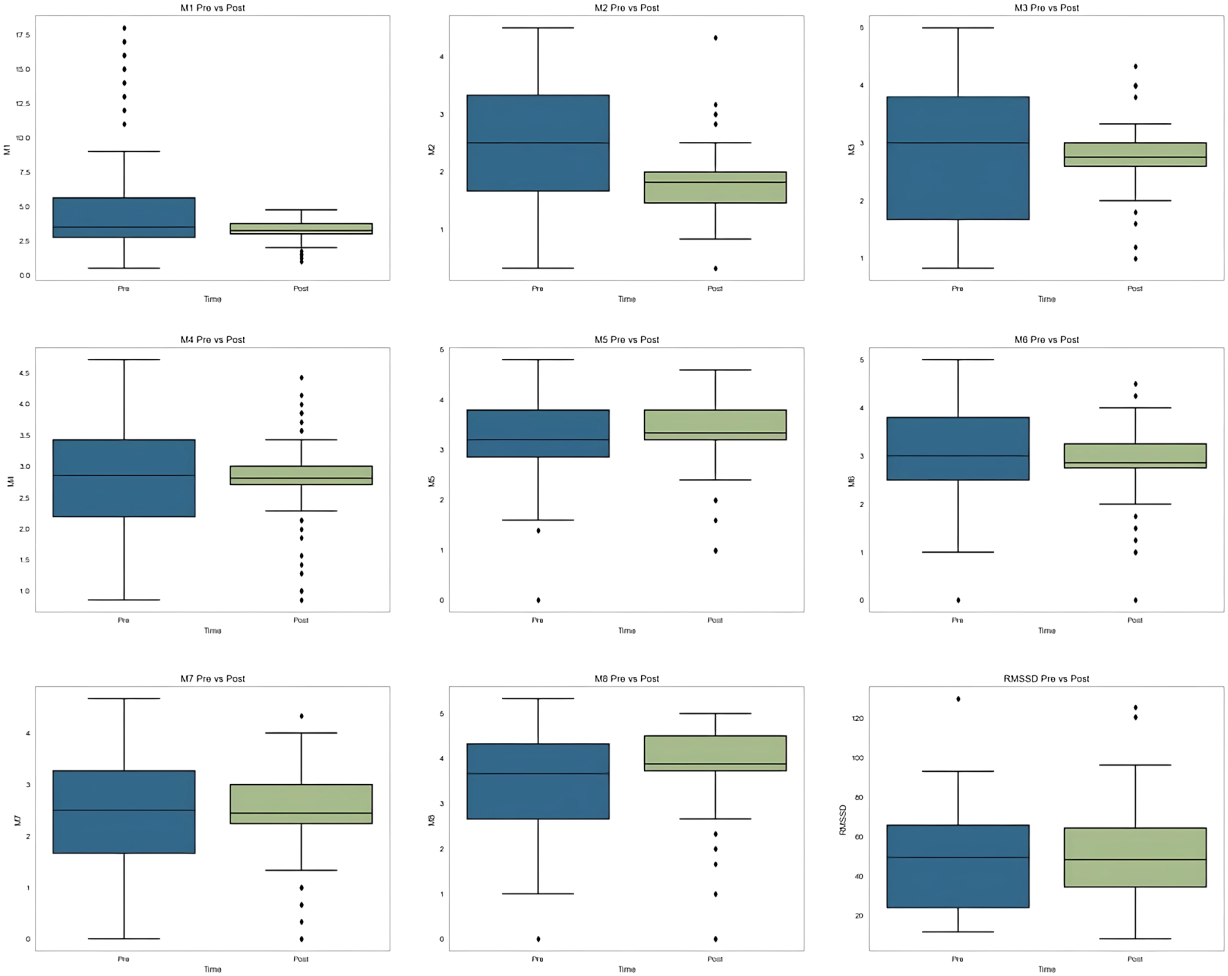
Change in interoceptive awareness sub‐scores and parasympathetic functioning among analogs. M1, noticing; M2, not distracting; M3, not worrying; M4, attention regulation; M5, emotional awareness; M6, self‐regulation; M7, body listening; M8, trusting; RMSSD, root mean square of successive differences between adjacent RR intervals.

Figure 5 reports the time evolution of interoceptive awareness and parasympathetic functioning during the different analog missions.

### 3.3 Adaptive labels

A PCA was performed on $M_{\text{delta}}$ based on data recorded at baseline and post‐mission. Three principal components with an eigenvalue greater than 1 were retained (Table 3).

**TABLE 3:** PCA loading matrix based on psychophysiological data.

Feature^a^	Factor 1	Factor 2	Factor 3
M1	**−0.603084**.	0.193812	0.292055
M2	**−0.492501**	0.014933	**0.331470**
M3	**0.308100**	**−**0.266251	**0.400273**
M4	**0.404623**	0.295373	0.144089
M5	0.111615	**0.437078**	0.083937
M6	**−**0.036385	**0.502032**	0.142423
M7	**−**0.020308	**0.553175**	**−0.449106**
M8	**0.333254**	0.223559	**0.532645**
RMSSD	**−**0.099166	0.070882	**0.333339**

*Note*: Bold values highlight the most significant correlations.

Abbreviations: M1, noticing; M2, not distracting; M3, not worrying; M4, attention regulation; M5, emotional awareness; M6, self‐regulation; M7, body listening, M8, trusting; RMSSD, root mean square of successive differences between adjacent RR intervals.

^a^ Correlations between each feature and the various factors are shown.

The first, which explained 45% of the variance, was weighted both negatively (*noticing*, *not‐distracting*), and positively (*not‐worrying*, *attention regulation*, *trusting*). This factor, which we named *the interoceptive perceptive response*, may indicate a disconnection of interoceptive perception that is characterized by both a decrease in initial levels of interoceptive perception, and a post‐mission increase in confidence in attention to low‐level interoceptive changes. Individuals with a positive coefficient have degraded interoceptive perception in terms of *noticing* and *not‐distracting*, but better functioning in terms of *not‐worrying*, *attention regulation*, and *trusting*, while the inverse also applies (a negative coefficient is consistent with improved *noticing* and *not‐distracting*, and degraded *not‐worrying*, *attention regulation* and *trusting*). This combination might be associated with the acceptance of negatively‐valued stimuli, and a reduced ability to anchor oneself in the present moment. Thus, this factor reflects an alteration in the first level of interoceptive processing, and conversely, greater confidence in attention to low‐level perceptual interoceptive changes.

The second factor explained 21% of the variance and concerned positive weightings given to *emotional awareness*, *self‐regulation*, and *body listening*. This factor, which we named *interoceptive body*–*mind integration*, may reflect reinforced integration of the highest levels of interoceptive information. Individuals with a positive coefficient on this factor increased their level of *emotional awareness*, *self‐regulation*, and *body listening*, and the inverse was also found. Therefore, a positive coefficient reflects more efficient processing and better integration of interoceptive information that strengthens mind–body integration and, thus, body awareness.

The third factor explained 8% of the variance and concerned positive weightings for *not‐distracting*, *not‐worrying*, and *trusting*, a negative weighting for *body listening*, and a positive weighting for RMSSD. This factor, which we named *the neuroceptive response*, may be understood as an unconscious perception of the level of safety induced by the ANS response. Individuals with a high coefficient on this factor score higher for *not‐distracting*, *not‐worrying*, and *trusting*, and parasympathetic functioning, but lower for *body listening*, and inversely. The body's reaction to a challenge may translate into an increase in the vagal system response and better interoceptive perception (except for *body listening*), as neuroception is an unconscious process.

The results of the PCA are presented in Table 3.

**FIGURE 6:**
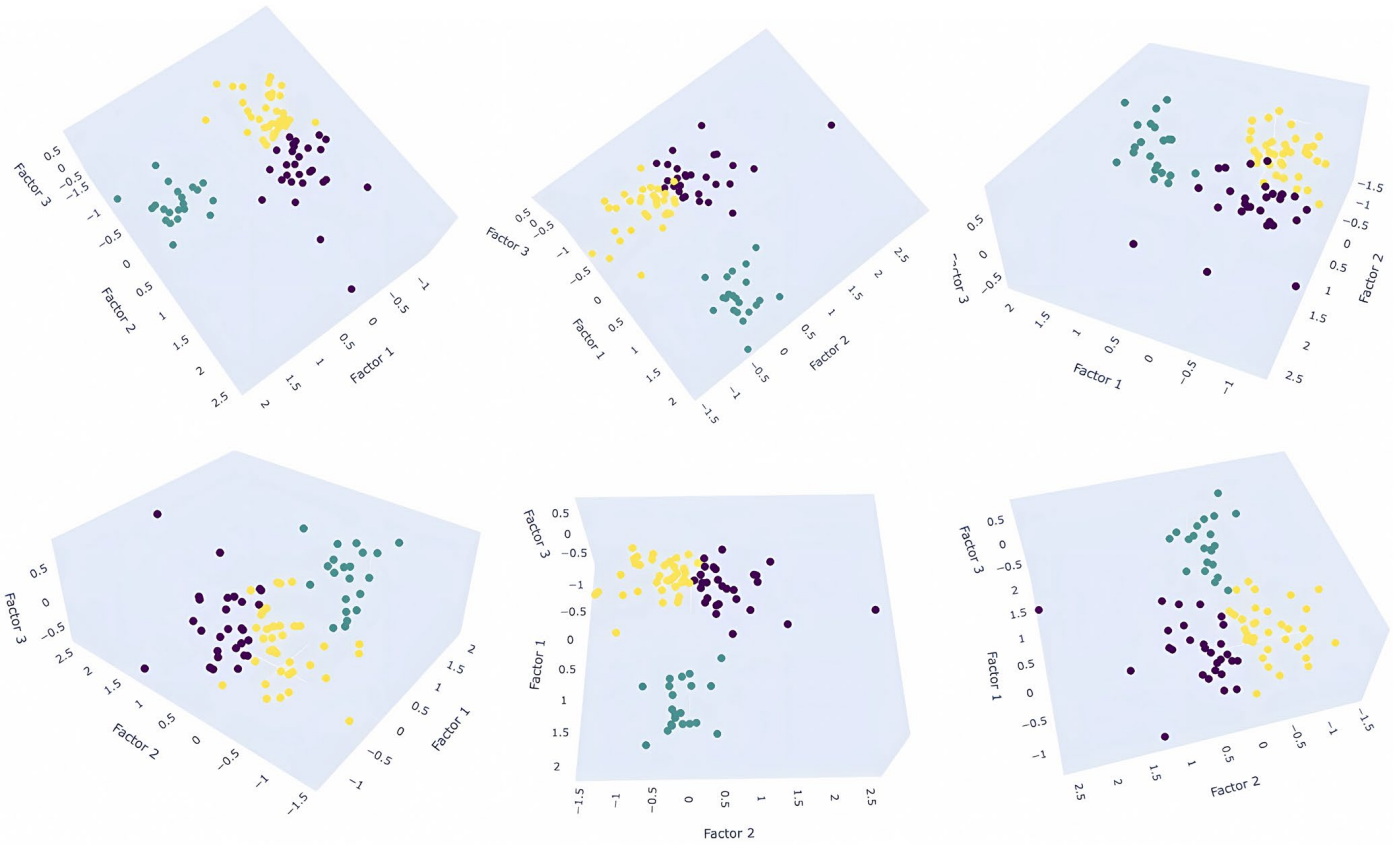
Distribution of participants based on *k*‐means clustering.

In the next step, a *k*‐means analysis was run on the overall variables used in the PCA analysis in order to create profiles of participants (Figure 6). The Elbow curve indicates an optimal partitioning with three profiles. No significant differences among profiles were found at baseline.

The yellow profile (class 0) included 35 participants (41.66% of the population). This profile scored negatively for factor 1, which targets the interoceptive perceptive response (there is one outlier with a positive value of 0.33), and for factor 2, which targets interoceptive body–mind integration. Scores varied widely for the third factor, ranging from −0.47 to 0.8 (14 negatives and 21 positives). Thus, this profile is characterized by a decrease in the first level of interoceptive perception and interoceptive body–mind integration (all levels of interoceptive awareness). However, the third factor does not discriminate this profile with respect to neuroception, although most participants scored positively.

The green profile (class 1) is composed of 21 participants (25% of the population). This profile scored positively for factor 1, which targets the interoceptive perceptive response, and negatively for factor 2, which targets interoceptive body–mind integration (except for four outliers who scored 0.12–0.47). Here again, scores varied widely for the third factor, ranging from −0.77 to 0.67 (12 negatives and 8 positives). Thus, this profile is characterized by increased confidence in attention to low‐level perceptual interoceptive changes, but decreased interoceptive body–mind integration. Like the yellow profile, the third factor cannot be used to discriminate this profile with respect to neuroception, although most participants scored negatively.

The purple profile (class 2) concerned 28 participants (33.33% of the population). This profile scored negatively for factor 1, which targets the interoceptive perceptive response (except for three outliers who scored 0.06, 0.32, and 0.36), and positively for factor 2, which targets interoceptive body–mind integration. This profile mainly scored negatively for the third factor, with values ranging from −0.68 to 0.66 (20 negatives and 9 positives). Thus, this profile is characterized by a decrease in the first level of interoceptive perception, but an increase in *body listening*, reflecting deteriorating interoceptive perception and increasing body–mind integration. Compared to the two previous profiles, the purple profile appears to impair neuroception, with gains in body listening.

Table 4 shows the distribution of space analogs for the three profiles identified by the *k*‐means analysis.

**TABLE 4:** Distribution of space analogs for the three profiles identified by the *k*‐means analysis.

Space analog	0	1	2
ENACT	11	‐	6
SSBN	10	‐	9
RAD'LÔ	‐	21	‐
ANTIDOTE	14	‐	13

Abbreviations: ANTIDOTE, simulation of a medical intervention following a CBRN attack while wearing protective equipment; ENACT, parabolic flight in microgravity; RAD'LÔ, simulation of survival at sea; SSBN, nuclear powered submarine.

### 3.4 Model performance and evaluation

**FIGURE 7:**
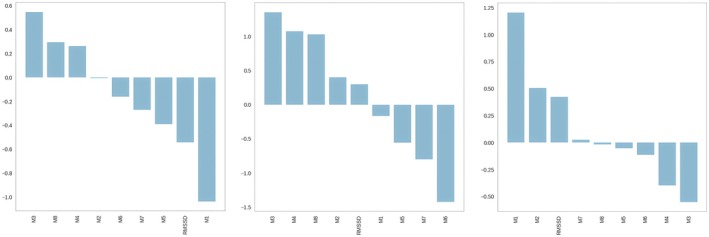
Importance of features obtained with the SVM method for distinguishing between each pair of classes. M1, noticing; M2, not distracting; M3, not worrying; M4, attention regulation; M5, emotional awareness; M6, self‐regulation; M7, body listening; M8, trusting; RMSSD, root mean square of successive differences between adjacent RR intervals.

Psychophysiological features recorded at baseline were used as input to train the SVM algorithm. The contribution of each feature, and the results of the *k*‐means analysis of baseline data were analyzed to identify which features best predicted $M_{\text{delta}}$. Figure 7 shows the importance of each feature for distinguishing between each pair of classes.

**FIGURE 8:**
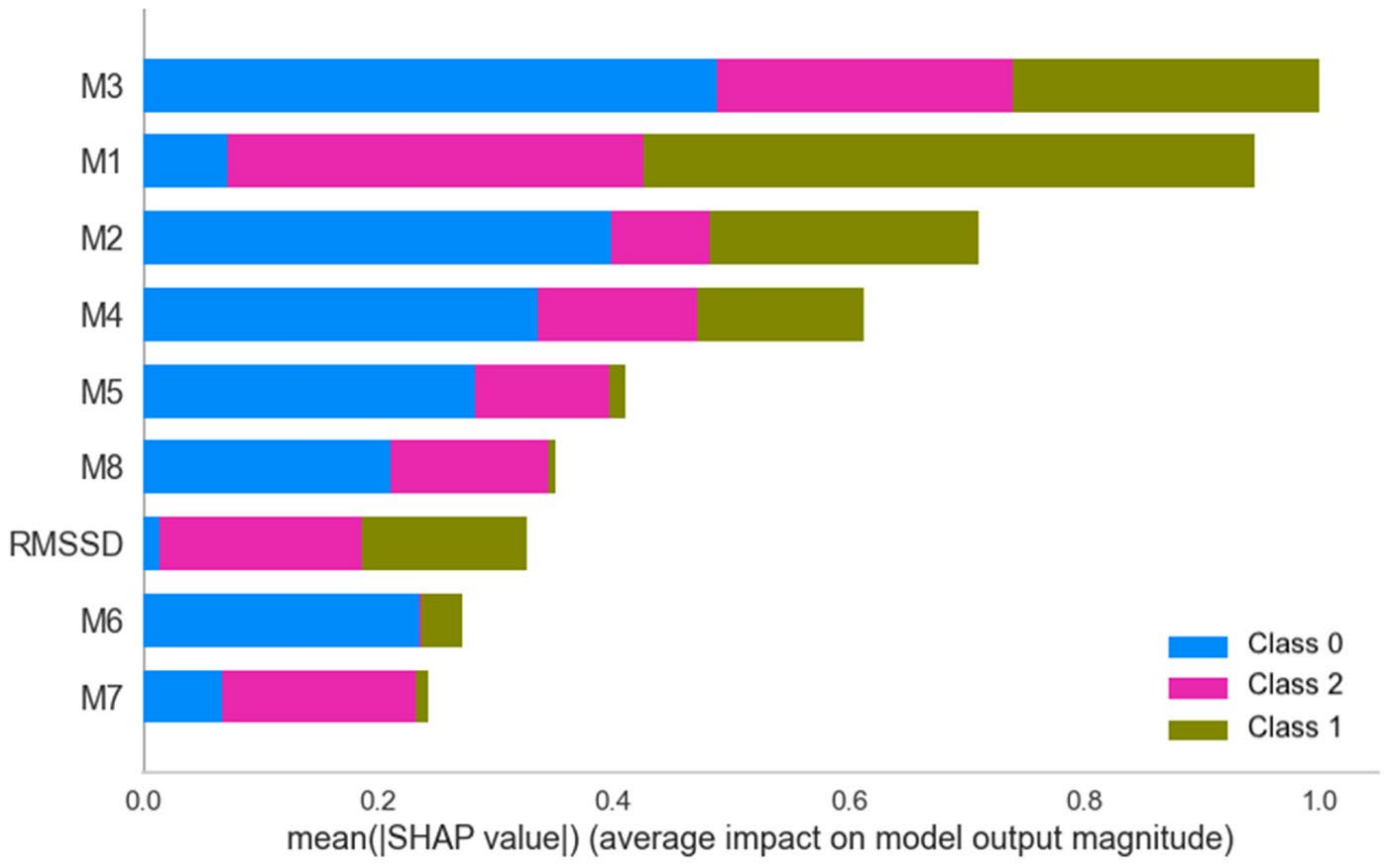
Importance of features obtained with the SHAP method for distinguishing between each pair of classes. M1, noticing; M2, not distracting; M3, not worrying; M4, attention regulation; M5, emotional awareness; M6, self‐regulation; M7, body listening; M8, trusting; RMSSD, root mean square of successive differences between adjacent RR intervals.

Shapley Additive Explanations (SHAP) are another valuable way to explore the contribution of each feature to the prediction of $M_{\text{delta}}$ (Figure 8). Both SVM coefficients and SHAP decomposition converge in identifying M1 and M3 as key adaptation class predictors. RMSSD was moderately important, suggesting that interoceptive markers may provide better class‐specific information than HRV alone.

**FIGURE 9:**
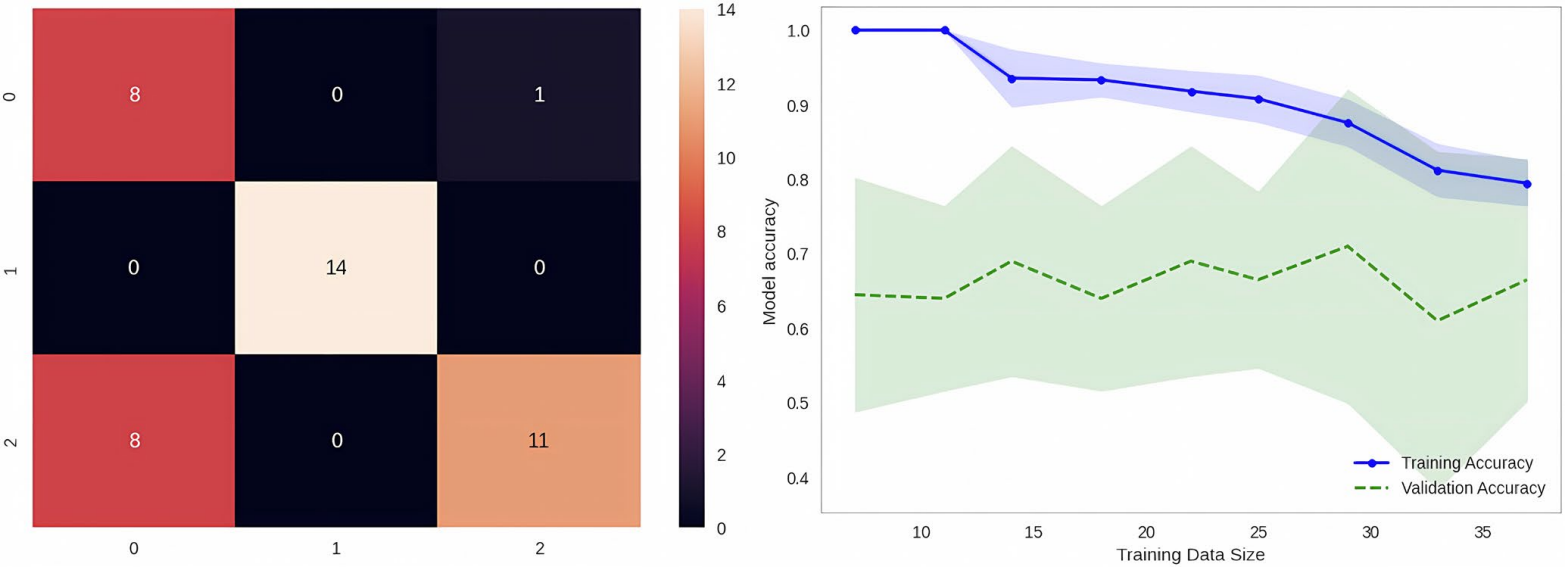
The confusion matrix and the learning curve.

SVM performance was 100% (specificity) and 79% (accuracy), based on the average model. The confusion matrix showed that almost all examples were correctly classified. The learning curve and the validation score seem to merge at the end, without reaching a clear convergence point (Figure 9).

Classification results obtained using the SVM method are reported in Table 5.

**TABLE 5:** Classification report.

Class	SEN (%)	F1‐score (%)	PRE (%)
0	0.89	0.64	0.50
1	1.00	1.00	1.00
2	0.58	0.71	0.92

Abbreviations: 0, yellow; 1, green; 2, purple; PRE, precision; SEN, sensitivity.

## 4 DISCUSSION

This study successfully applied machine learning techniques to predict post‐mission adaptation profiles based on pre‐mission psychological and physiological states. In addition to risk exposure, which has been confirmed as a key factor in previous studies for example, (Le Roy, Aufauvre‐Poupon, et al., 2023; Palinkas & Suedfeld, 2021), it is necessary to identify crew members who will have the poorest adaptive response. LDSE astronauts will face many challenges that may compromise the success of the mission. Thus, there is a need to implement new solutions that can evaluate the risks they will face and improve their health before departure.

### 4.1 Adaptation profiles and space analog missions

Our study highlights three response profiles. A PCA comparison of psychophysiological features on return, compared to the day before the start of the mission, underlined three factors. Unexpectedly, the interoceptive sub‐factors *not‐worrying* and *trusting* were identified in both factors 1 and 3. This result may be explained in two ways. First, two versions of the MAIA questionnaire were used in the studies that composed our dataset. Mehling and colleagues initially developed a version in 2012 (Mehling et al., 2012), and then a second in 2018 (Da Costa Silva et al., 2022; Mehling et al., 2018) to improve the quality of the scale. The *not‐worrying* sub‐factor was one of those modified in the new version of the questionnaire, potentially introducing a bias into our results. Furthermore, the *trusting* sub‐factor appears to be decorrelated from the other MAIA dimensions. A recent study showed an association between the severity of depression and loss of trust in bodily sensations (Dunne et al., 2021). The latter authors argued that a reduction in *body trusting* could subsequently initiate change in interoceptive processes.

Thus, the positive component of factor 3 might translate into enhanced neuroception, and inversely for its negative component. Neuroception involves the perception of safety/insecurity in the environment through the ANS, as described by Porges (Porges, 2004). This neural process is exclusively unconscious. The negative axis for *body listening* supports this line of reasoning, as it implies a conversely conscious process of listening to the body. RMSSD is considered to be a brake on stress and thus a marker of stress vulnerability (Beauchaine & Thayer, 2015; Le Roy, Aufauvre‐Poupon, et al., 2023; Thayer et al., 2012). This type of response is important when environmental constraints are sufficient to trigger adaptive behavior.

The factors identified in the PCA were used in a *k*‐means clustering analysis to determine adaptation profiles. Individuals in the yellow group have impaired processing of all levels of interoceptive information. Other authors (Mehling et al., 2012; Mehling et al., 2018) have distinguished four levels of body awareness: (1) a first perceptual and sensory level, which involves the perception of sensations and the ability to discern bodily cues that provide information about the person's physiological and emotional state; (2) a second level, in which attention is directed towards bodily sensations and the attentional response is coupled with an emotional evaluation; (3) a third level, in which interoceptive awareness corresponds to beliefs relating to bodily sensations; and (4) a fourth level, in which body–mind integration corresponds to holistic body awareness.

The interoceptive system could be responsible for creating homeostatic maps of the body that serve to orchestrate regulatory responses over time (Damasio & Carvalho, 2013). Significant inter‐individual variability in the neuroceptive response was found in the yellow group, and there is evidence of interoceptive dysfunction in coping with mission constraints in this group. This dysfunction appears to be associated with an improvement in neuroception (for some) or a deterioration (for others). The latter group may evolve towards a situation in which neuroception increases insecurity as the environment becomes more stressful.

A disconnect in interoceptive perception is observed among individuals in the green group. This is evidenced by increased reliance on attention to low‐level perceptual interoceptive changes and impaired processing of high‐level interoceptive information. As with the yellow profile, there was considerable inter‐individual variability in neuroceptive responses, but here, its loss is evident among a higher percentage of individuals.

A disconnect in interoceptive perception is also observed among individuals in the purple group. This is evidenced by an impairment in the first level of interoceptive processing and an improvement in mind–body integration. Moreover, many individuals exhibited impaired neuroception (71.42%). Nevertheless, this is the only profile where there is evidence of physiological adaptation, measured as high parasympathetic functioning (RMSSD). This result illustrates the close link between psychological and physiological neuropathways leading to allostasis and the importance of measuring them both to better understand the adaptive (or maladaptive) response of individuals who are subjected to stressful environments.

### 4.2 Distribution of adaptation profiles in space analogs

One important result concerns the distribution of adaptation profiles in different space analog environments.

The green profile is exclusively composed of participants in the RAD'LÔ study, while a similar distribution of individuals with yellow and purple profiles is seen in ENACT, SSBN, and ANTIDOTE studies. Survival at sea appears to be the environment that is most stressful, and it has been described as one of the most complex survival situations (Field Manual, 1992), with the most powerful stressors (Sarinas & Tagulalap, 2017; Motley, 2005). In related work, Chen and colleagues (Chen et al., 2016) studied the adaptation of overwinterers at several polar stations. They found that the more severe the environmental characteristics, the more deleterious the effects. The response to survival at sea is associated with bodily dissociation and heightened hypersensitivity to stimuli in the face of an overstimulating sensory environment (Le Roy, Martin‐Krumm, et al., 2024). It appears that the more extreme the environment, the greater the environmental demand, and the more complex the adaptation to cope with these stressors.

Research into preventive and anticipatory countermeasures is therefore fundamental to ensuring health under stress. Countermeasures intervene before the emergence of pathology, to prevent its occurrence, or delay or limit its effects. In the case of LDSE, the concern is not to eliminate the emergence of a disease, but to ensure appropriate, early‐phase treatment and support an adaptive human response. Two other challenges are: (1) to understand the mechanisms by which the individual's resources are no longer sufficient to ensure his or her adaptation to the environment; or, alternatively, (2) to understand why some individuals are able to maintain their adaptive capacities when exposed to the same level of stressors.

Overall, our study confirms the importance of offering specific support to maintain operational capacity. Crew members who are less‐able to adapt should receive targeted training. These programs need to be carefully adjusted to each individual's ability to adapt their profile and/or the environment to which they will be exposed, particularly when this involves high‐intensity stressors. How interoceptive information is processed seems to be a useful way to classify crew members during a space analog mission, and supports the idea that interoceptive pathways are involved in adaptation. Our results confirm previous findings that associate interoception with mental health (Herbert et al., 2021), and suggest that it may be beneficial in managing stress responses in the long‐term (Beauchaine & Thayer, 2015; Porges, 1995).

### 4.3 Predicting adaptation profiles using machine learning

Our results from the machine learning model capture this complexity. The SVM model was able to predict the three interoceptive‐physiological response profiles from different space analogs, using baseline measures of psychophysiological features. No difference was observed between the populations pre‐departure. While it is clear that individuals are carefully selected before they are sent to these unusual environments, using similar selection processes, it is difficult to identify one profile that will be optimally adapted, and another that will become increasingly vulnerable over the course of a mission.

A recent review (Le Roy, Martin‐Krumm, et al., 2023) discussed the lack of a consensus regarding the psycho‐cognitive mechanisms underlying adaptation, and noted that this is probably due to its multifactorial nature, significant inter‐individual variation, and inter‐study bias. Stress is a complex state, and information from multimodal sources is required to reliably assess both vulnerability and adaptation. The SVM method is considered one of the best classification techniques (Schultebraucks & Galatzer‐Levy, 2019; Sharma & Gedeon, 2012), particularly in the case of small samples (Zhang et al., 2019), and it is known to be an effective classifier for assessing physiological data derived from cardiac biosignals (Pluntke et al., 2019; Vanitha & Suresh, 2014). However, to the best of our knowledge, it has rarely been used to investigate interoceptive sensitivity, despite the fact that it is highly accurate, and requires little computational power.

The classification report shows that the model performs well for the green profile, achieving high sensitivity, F1‐score, and precision. For the purple profile, it performed well for precision and F1‐score, but sensitivity was poorer than for the yellow profile. The report provides a comprehensive view of the model's performance for each class, considering both false positives and false negatives. However, validation accuracy fluctuated. This behavior suggests that the model's performance is not stable enough, and might be sensitive to variation in the validation dataset (due to, for example, noise or outliers). Nevertheless, the split into a training and a test dataset is useful in the case of a small sample (Vabalas et al., 2019).

The learning curve may reflect an unrepresentative dataset. The latter may not capture the statistical characteristics of both the training and the validation dataset, and fail to provide sufficient information to learn the classification problem. In our study, the 79% accuracy achieved by the SVM may be explained by the difficulty of accounting for inter‐individual differences, and the effect may be exacerbated by our small sample size (although our sample is large in the context of space analog studies). Of our 84 participants, only 42 were included in each training and validation dataset.

Furthermore, the quality of the ECG signal can impact classification performance, as a noisy signal can reduce accuracy (Zhang et al., 2019). Signal entropy can introduce redundant or overlapping information, which impairs performance (even though features in the non‐linear domain are more robust than those derived from the linear domain). A linear kernel SVM classifier can model these types of variables more naturally as it uses a small number of free parameters. Conversely, SVM performance is improved when the boundary between the two classes is unclear (and weakened when the boundary is clear). As vectors furthest from the hyperplane have more obvious class labels, the SVM can calculate the hyperplane separation between the two classes more accurately. Recent research highlights that stress detection models based on physiological features perform better in controlled laboratory conditions than in an ecological context, a result that was consistent among the included studies (Mishra et al., 2020). Thus, our model may not generalize well to data from unseen participants due to high inter‐individual variability, as already highlighted in the literature (Le Roy, Martin‐Krumm, et al., 2023).

Further research is needed, and we will pursue our attempts to more accurately predict adaptation profiles. Given the possible biases inherent in the data collection environment, as well as the limited number of training examples, our results are promising. While our algorithm may be used as an initial triage tool, it should not be used as a substitute for screening by a medical team, nor replace pre‐departure screening at this time.

Another avenue for future research is to identify the variables that account for the largest amount of variance. Our profiles were able to identify the best‐adapted and the most vulnerable individuals. Our work represents a first step in using a machine learning algorithm to identify predictors of adaptation during a space analog mission. If confirmed, our results suggest that several approaches and countermeasures should be studied to improve the health of astronauts involved in future LDSE missions. Importantly, the ability of the AI model to predict adaptation profiles from pre‐mission data supports its potential as a screening tool for astronaut selection. Moreover, if integrated into wearable monitoring systems, this approach could enable real‐time monitoring of crew health during space missions, and facilitate the timely deployment of countermeasures. These findings could support individualized training regimens or selection tools based on interoceptive profiles. With further validation, these models could also contribute to real‐time health monitoring during missions.

## 5 LIMITATIONS

Our study has several major limitations. The first is the small sample size, and an imbalance between male and female participants, and right‐ and left‐handed individuals. Studying such a population is complex, both in terms of time constraints, and access to infrastructure and personnel (i.e., operational constraints, attendance). Our adaptation profiles reflect the reality of the field and the challenges of the mission. Both the scientific team and participants must be flexible to run experiments in space analog environments. Our results need to be confirmed with a larger dataset, which could be collected over several years (for example, in the context of a doctoral research project) as time constraints limited our corpus of recorded measures.

Secondly, our results are not reproducible beyond the specific experimental conditions and cannot be generalized. Our space analogs were atypical, isolated, and confined, as well as extreme and unusual environments with specific stressors. Thirdly, this study is an example of a binary classification problem. Adaptation in a multifactorial and multi‐class problem might be a better way to predict change based on psychophysiological functioning before and after the mission. Fourth, the use of the MAIA questionnaire limits the subjective assessment of individuals' interoceptive sensitivity. Although the latest version seems promising, a more objective assessment of the integration of interoceptive information could have improved profile discrimination. Moreover, two different versions of the questionnaire were used. Intelligent sensors would provide more objective measures of participants' adaptation to extreme environments. The use of a tool to evaluate emotional regulation would be informative regarding its involvement in interoception and the ANS response (Le Roy, Giaume, et al., 2024). Fifth, different ECG devices were used to record RR intervals. The use of a single, gold‐standard ECG system may improve inter‐individual data reliability.

## 6 CONCLUSION

The present study is one of the first to test the ability of AI to predict psychophysiological adaptation in space analog environments from baseline features. The theoretical framework is based on interoceptive‐vagal integration, which is recognized as an important model of adaptation to stress. Various space analog environments were investigated to assess the possibility of extrapolating the results to actual space missions. Our findings reflect the complex, multifactorial nature of adaptation. They suggest a modification in the processing of interoceptive information over the course of the mission, as well as the modulation of vagal ANS responses.

Given the difficulty of conducting work in the field, it is essential that this research into identifying the most predictive variables under stress continues, and this factor should be included as a modality in machine learning models. Also, our results show that although selection processes are similar, and designed to assess the person's ability to adapt to a constrained environment, it is difficult to predict adaptive capacities on an individual level. Only by studying adaptation mechanisms can efficient countermeasures be established to safeguard crew health.

By harnessing the power of AI to decode the hidden dynamics of human adaptation, this study, despite many limitations, marks a step forward in preparing for the physiological and psychological demands of space exploration—where understanding stress responses is not a luxury, but a necessity for survival.

## FUNDING INFORMATION

This work was supported by the French Space Agency (CNES, n°4800001159) and the French Military Health Service.

## CONFLICT OF INTEREST STATEMENT

The authors declare that the research was conducted in the absence of any commercial or financial relationships that could be construed as a potential conflict of interest.

## ETHICS STATEMENT

This research uses data from experiments conducted in four space analogs, and approved by a Committee for the Protection of Individuals. The first (ENACT) concerns parabolic flight in microgravity, an extreme and unusual environment (EUE) (ID‐RCB: 2022‐A01317‐36, Committee for the protection of individuals Ile‐de‐France XI). The second concerns a two‐month mission in an SSBN, an isolated and confined environment (ICE) (ID‐RCB: 2017‐A01329‐44, Committee for the protection of individuals Sud‐Est VI). The third (RAD'LÔ) concerns a simulation of survival at sea (ID‐RCB: 2017‐A01329‐44, Committee for the protection of individuals Sud‐Est VI), and the fourth (ANTIDOTE) concerns a medical intervention following a simulated CBRN attack. In the latter case, participants wore protective equipment that could be compared to an astronaut's extravehicular suit (ID‐RCB: 2021‐A03057‐34, Committee for the protection of individuals Ile‐de‐France XI).

## CONSENT

Written consent was obtained for each study.

## Data Availability

Data collected are confidential. Any availability must be request from the Admiral of the Strategic Oceanic Forces. Please contact marion.trousselard@gmail.com for the request.
